# A Radiographic Technique for Assessment of Morphologic Variations of the Equine Caudal Cervical Spine

**DOI:** 10.3390/ani10040667

**Published:** 2020-04-12

**Authors:** Christine Gee, Alison Small, Kathleen Shorter, Wendy Y. Brown

**Affiliations:** 1 Canine and Equine Research Group, University of New England, Armidale 2351, NSW, Australia; alison.small@csiro.au (A.S.); kshorter@une.edu.au (K.S.); 2 Agriculture and Food, CSIRO, New England Highway, Armidale 2350, NSW, Australia

**Keywords:** C6 malformation, horse, morphologic variation, neck, pain-based behavior, spine, vertebrae, x-ray

## Abstract

**Simple Summary:**

Equine Caudal Cervical Morphological Variation (ECCMV) has been acknowledged as a common finding in horses. The equine sixth cervical vertebrae (C6) with two caudal ventral laminae appears to be prone to the most congenital variation. The direct clinical relevance of this condition is currently unknown. A standardized method of assessment and diagnosis in live horses would allow for scientifically robust, quantitative studies into the relevance of this syndrome. Equine radiographic imaging is standard within veterinary practice and offers an accessible screening and diagnostic tool for veterinarians and provides a starting point for assessment. Here, we present a systematic method for locating cervical landmarks and radiographing with specific views as a reliable, repeatable imaging procedure for identifying ECCMV. If ECCMV is linked to poor performance, pain-based behaviors, lameness, and horse and rider safety and welfare, then a protocol for testing and diagnosis could be vital to improving horse rehabilitation, management, and wellness.

**Abstract:**

Equine Caudal Cervical Morphologic Variation (ECCMV) is a congenital malformation of the caudal cervical spine distinct from the more commonly recognized Cervical Vertebral Stenotic Myelopathy (CVSM). The most common presentation of ECCMV is recognized on the sixth cervical vertebra (C6). In “normal” presentations, the transverse processes on the left and right sides have a caudal lamina projecting ventrally below the caudal vertebral body in a heel shape. With ECCMV, variations occur to the structure of the caudal ventral lamina on one or both sides of C6 and may include the seventh cervical (C7) and first thoracic (T1) vertebrae and ribs, in varying configurations. Whereas the prevalence of ECCMV is not known, it has been recognized for many years and has been reported to occur with relatively high frequency within multiple populations of domesticated horses. To date, there is no documented link between the occurrence of ECCMV and clinical signs. However, based on retrospective studies, multiple authors have recognized the potential impact on performance that this condition may have. Establishing a reliable radiographic protocol for the consistent diagnosis of ECCMV would allow quantitative, scientific evaluation of the problem and support clinicians working in this field. We present a radiographic technique, which has been illustrated by diagnosis of ECCMV in three horses and confirmation of the diagnoses in two cases via postmortem examination.

## 1. Introduction

Equine Caudal Cervical Morphologic Variations (ECCMV) are congenital malformations of vertebrae in the caudal cervical and cranial thoracic spine. Morphologic variations have been identified in specific bony and soft tissue structures: Sixth cervical vertebra (C6) and seventh cervical vertebra (C7), first and second sternal ribs, musculature (*m. longus colli* and *m. scalenus ventralis*), and nerves (phrenic nerve and brachial plexus) [1−3]. The principle anomaly is the altered shape of the normal ventral lamina that is unique to C6, and this is the only skeletal constant in all cases. Unilateral and bilateral presentations have been identified. These categories describe the symmetrical (bilateral) or asymmetrical (unilateral) absence (or altered shape) of the ventral lamina of the transverse process. Possible combinations include: C6 only; C6 and C7; C6, C7, and first sternal rib; C6, C7, first and second sternal rib [1,2]. These presentations begin with alterations to the ventral lamina at C6 and then involve varying osseous (and soft tissue) changes in progressive steps through the successive vertebrae and ribs. Morphologic variations occurring secondary to laminae absence include variable shapes and sizes of the laminae, transposition of the laminae onto caudal vertebrae, altered arterial foramina, lack of symmetry of vertebrae, altered facet joint symmetry, vertebral canal asymmetry, and rib malformations [2].

The heritability of these morphological variations have not been directly investigated to date [3]. Studies have shown that ECCMV is not breed-specific, with the identification of ECCMV in Thoroughbreds, Warmbloods, Arabians, Standardbreds, Quarter Horses, and associated derivatives [2,4,5]. In addition, ECCMV is not geographically isolated, with studies identifying the congenital malformation in Europe, USA, UK, Australia, and Japan, with one study reporting a prevalence of 38% in Australian Thoroughbreds [2] and another demonstrating 34% in Warmbloods [4].

Variations in musculature have been identified in ECCMV cases along with anomalous nerve pathways. The *m. longus colli* (thoracic portion) exhibits the most significant changes in horses with C6 morphological variations [3]. Rombach et al. note that the short, deep muscles of the cervical spine provide dynamic segmental stability and support to the individual vertebral joints, as well as postural control when working synergistically with *m. longus capitus* [6]. *M. longus colli* continues as a fusiform muscle belly, with *m. longus thoracis* extending from the ventral transverse process of C6 to the T5–T6. Its attachment to C6 at the ventromedial aspect of the transverse process is via a strong tendon, which supports the ventral aspect of the vertebral spine in the cervicothoracic region [6]. As Rombach stated, “Effective stabilization by the deep paravertebral muscles may reduce the risk of osteoarthritis and development of neck pain in horses, which is a limiting factor for performance of such animals” [6]. De Rouen [4] noted that morphological variations in the C6 ventral laminae may be linked to other abnormalities affecting regional biomechanics and should be considered clinically relevant: “The alteration of the attachment site for regional musculature due to anomalous C6 may lead to altered biomechanical forces resulting in perceived pain or reduced range of motion” [4]. A conclusion was made that these horses may be experiencing *dynamic* pain or pain from conditions not detected by static or plain radiography. These observations suggest that identification of these horses and further investigation into the clinical relevance of this syndrome is warranted. It should also be noted that dynamic pain is difficult to elicit and assess under in hand veterinary neurological testing compared to pain that may manifest under saddle, at speed and over jumps.

Previous studies into the clinical relevance of ECCMV have been retrospective [4,7,8] and, in some cases, have involved multiple examiners and locations [4,8,9]. In 2018, Dyson et al. found increasing evidence that nerve root injury may cause forelimb lameness, and 3 of the 25 horses in the study had overt ECCMV [4,8,9]. Veraa et al. found no correlation between morphological variation and clinical signs in Warmblood horses. However, the control groups were younger, higher price-range horses, which were presented for pre-purchase examination (less likely to be symptomatic), and affected horses had exhibited long-standing clinical signs [8]. Subjectivity of the neurological examination and detection of subtle clinical signs pose further challenges to diagnosing this disorder. If horses with altered lower cervical biomechanics are more prone to degenerative changes and pain as suggested by Rombach [6], then the progression of the syndrome over time may well be relevant. This has implications for both horse welfare and rider safety, as horses affected by this condition have been shown to exhibit lameness, pain-based behaviors, and failure to perform to expectation [9].

The manifestations of ECCMV are poorly understood due to the complexity of local anatomy and difficulty in obtaining a complete diagnostic evaluation. Standard veterinary neurological and clinical examinations may well miss what is suspected to be an intermittent and dynamic instability in some cases [4,8,9]. For these reasons, there is a high likelihood that ECCMV is frequently under diagnosed in veterinary practice. Diagnosable conditions affecting performance have broader implications for the equine and equestrian industries in general.

The research aim was to develop an objective assessment or screening technique for ECCMV. Three cases are presented, with the permission of the horse owners, to illustrate the radiographic technique, including two horses for which postmortem results were available.

## 2. Materials and Methods

### 2.1. Study Animals and History

The three cases demonstrating this radiographic technique were horses being investigated for cervical-based pain. Horse F1 was an eight-year-old Australian Stock Horse mare. Horse M1 was a ten-year-old Australian Stock Horse gelding. Horse M2 a six-year-old Warmblood gelding.

### 2.2. Orientation

**Figure 1:**
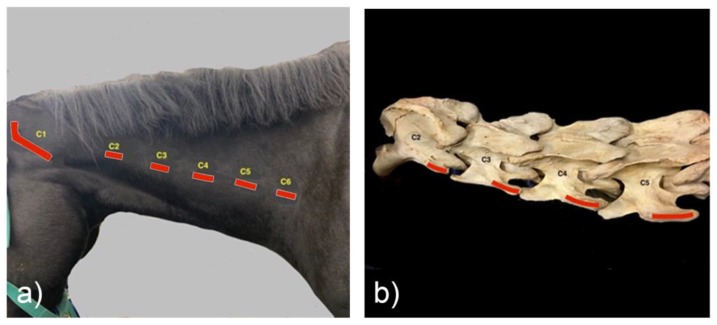
(**a**) Lateral neck with palpable landmarks of the caudal transverse processes marked in relation to the cervical vertebrae (C1–C6). (**b**) Equine cervical vertebrae C2 to C5, with red lines indicating palpable landmarks.

Standing on the left side of the horse, with the clinician’s right hand parallel to the vertebrae, fingers pointing cranially, with a gentle calcaneal contact, the transverse processes (TPs) can be located. The fingers locate the lateral wing of the atlas or C1. Travelling caudo-ventrally, the first palpable small bony prominence is the reduced, caudal TP of the axis, C2 (Figure 1). Continuing to travel with the heel of the hand, the next most lateral bony prominence is the TP of C3, then C4, and then C5. The transverse process is a wide, thin structure horizontally oriented and, in a standard 500-kg horse, is approximately 2–3 cm in length on palpation.

For repetition, a radiodense marker was placed approximately 5 cm dorsal to the transverse process of C5. The TP of C6 is generally the last palpable landmark in the cervical spine in standing horses.

### 2.3. Radiographic Technique

Intravenous standing sedation with Detomidine Hydrochloride (Detomidine Hydrochloride 10 mg/mL—Virbac) was necessary for image capture, with a dose rate on average of 0.08 mL/100 kg. Images were taken in a standing position with a straight cervical spine from the external occipital protuberance through the upper thoracics to the lumbosacral joint, with manual support of the head if required. The patient stood with neutral head height and positioning of the patient’s forelimbs behind the vertical. Cranial traction, applied to the patient’s mandibular rami, assisted in forward extension of the cervical vertebrae cranial to the shoulder articulation, which can obscure C6 and C7 in some horses.

**Figure 2:**
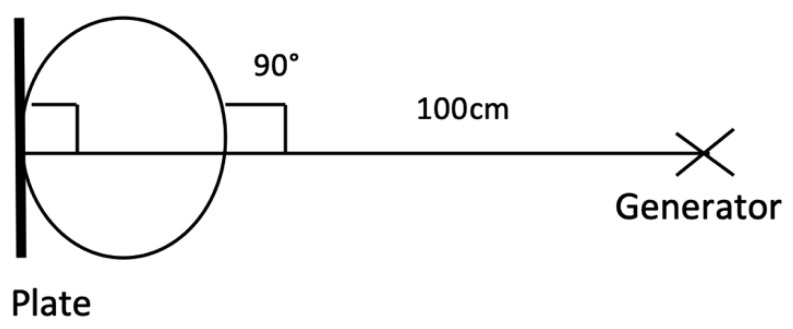
Standardized laterolateral positioning for radiographs.

Radiographs were carried out utilizing digital radiographic (DR) systems and veterinary image management software with the same examiner present at all procedures (CG). Generators with outputs of 90–100 KV and 20–30 mA were used. Examples of exposures are: Upper cervical; 80 KV, 2 mAs, mid cervical; 84 KV, 2.5 mAs, lower cervical; 90 KV, 3.0 mAs. Upper cervical images were taken in the laterolateral projection with a film focal distance (FFD) of 100 cm (Figure 2).

**Figure 3:**
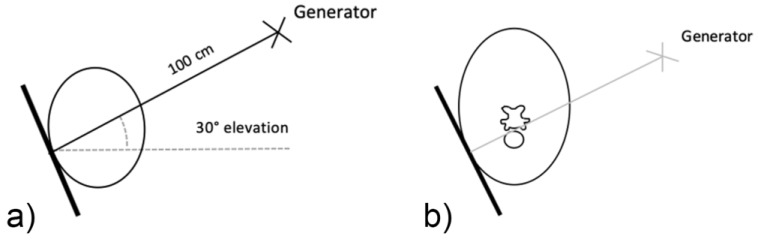
Lateral 30° dorsal-ventral oblique view, left and right side required (**a**). Ideal positioning for image capture and superimposition of the C6 ventral lamina over the trachea (**b**).

**Figure 4:**
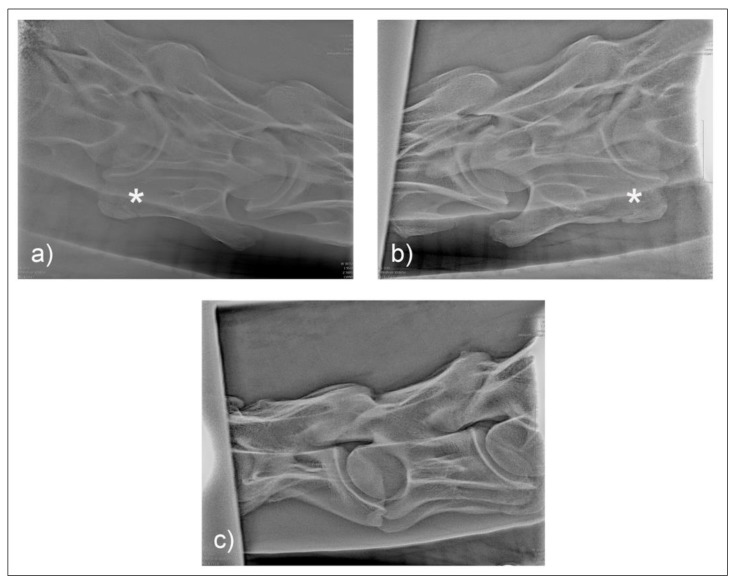
Normal radiographic appearance of C6 with 30° dorsal to ventral oblique right (**a**) and left (**b**). In the laterolateral view (**c**), the head is to the left, and both left and right ventral laminae are visible. Note the incomplete centers of ossification of the ventral laminae (*). (Images have been left in nonstandard orientation for simplicity).

For the first plate, the beam was centered on the first and second cervical vertebral articulation (C1 and C2) in a laterolateral orientation with 0° of elevation. The standard laterolateral views have been described by many authors [7,10,11]. A lateral 30° dorsal to ventral oblique highlights the lamina of C6 against the radiolucent trachea (Figure 3). Figure 4 illustrates the normal radiographic appearance of C6 using this technique.

### 2.4. Postmortem Examination

Field autopsies to assess the osseous changes were carried out in order to confirm the radiographic diagnosis of ECCMV in F1 and M1. The cervical and cranial thoracic vertebrae of M1 and cervical vertebrae and cranial bones of F1 were then boiled and cleaned for further examination.

## 3. Results

**Figure 5:**
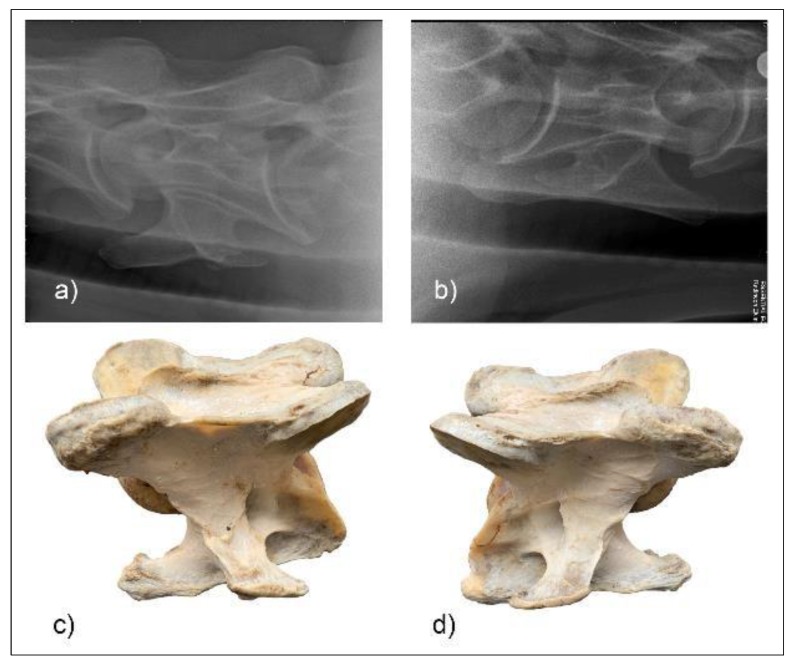
Radiographic images of the lateral 30° dorsal to ventral oblique left (**a**) and right (**b**) of C6 of horse M1 with the vertebrae left (**c**) and right (**d**) below. (Image (**b**) and (**d**) have been left in nonstandard orientation to appreciate the three-dimensional osseous form).

**Figure 6:**
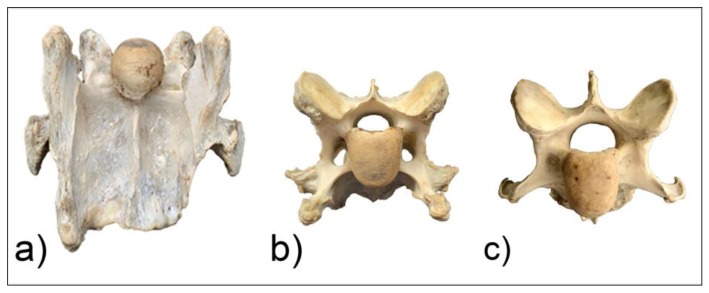
The ventral aspect of C6 (**a**) and cranial views of C6 (**b**) and C7 (**c**) of horse M1.

**Figure 7:**
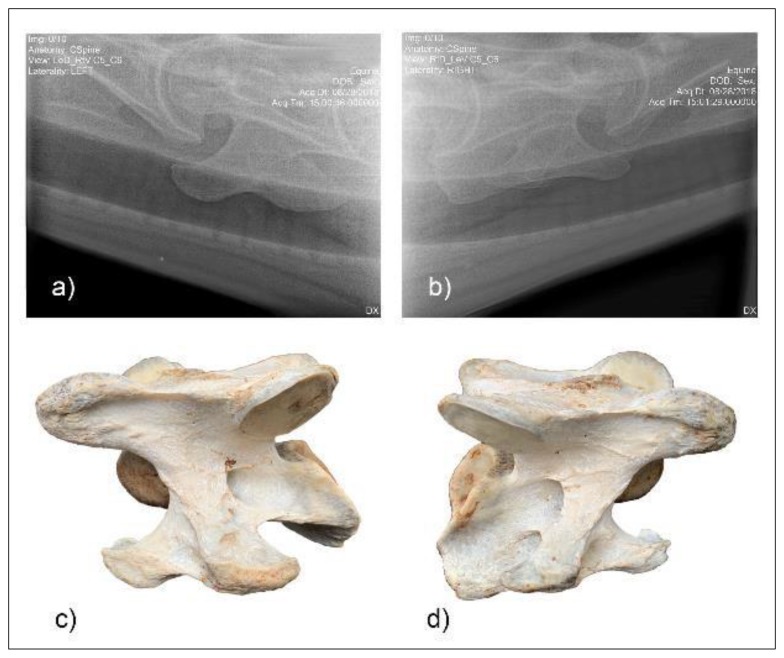
Radiographic images of the lateral 30° dorsal to ventral oblique left (**a**) and right (**b**) of C6 of horse F1 with the vertebrae left (**c**) and right (**b**) below. (Image (**b**) and (**d**) have been left in nonstandard orientation to appreciate the three-dimensional osseous form).

**Figure 8:**
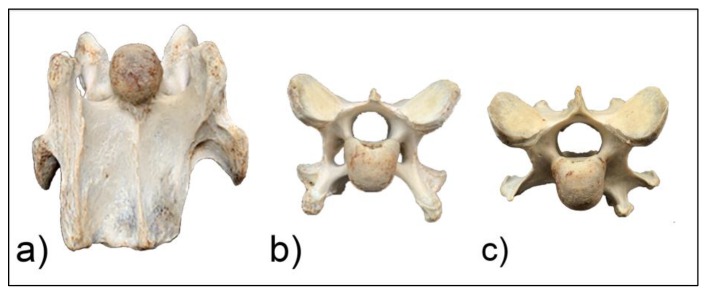
The ventral aspect of C6 (**a**) and cranial views of C6 (**b**) and C7 (**c**) of horse F1.

**Figure 9:**
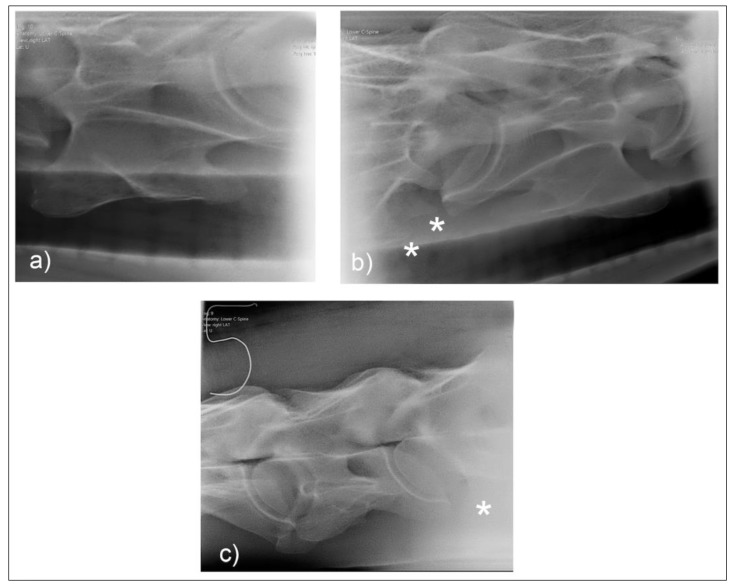
The radiographic images of the 30° dorsal to ventral oblique left (**a**) and right (**b**) of C6 in horse M2 and the laterolateral view (**c**) of horse M2 with bilateral morphologic variation of C6 and transposition on to C7 (*). (Note example of marker dorsal to C5 transverse process).

Two horses (F1, M1) presented with unilateral morphological variations of C6. In horse F1, C7 was also affected, which has been noted to be more common in females [12]. Postmortem examination of horses M1 (Figure 5 and Figure 6) and F1 (Figure 7 and Figure 8) revealed morphological variations consistent with radiographic images and confirming the diagnosis of ECCMV in both cases. The remaining horse (M2) presented with a symmetrical bilateral variation of C6 (Figure 9), with transposition on to C7 visible on the radiographs (Figure 9c). The morphological variations can be appreciated in the ventral and cranial views of the bones, as well as the 30° oblique views (Figure 6 and Figure 8).

On postmortem examination of horse M1, there was no gross transposition of the missing left caudal lamina on to the C7 vertebrae. There was, however, a small spur 2 mm from the ventral midline of the mid vertebral body on C7, which was otherwise symmetrical.

On postmortem examination of F1, the absent left caudal lamina of C6 was transposed onto the ventrolateral aspect of C7 creating a gross left to right asymmetry in C7 as well as the C6 vertebrae.

**Figure 10:**
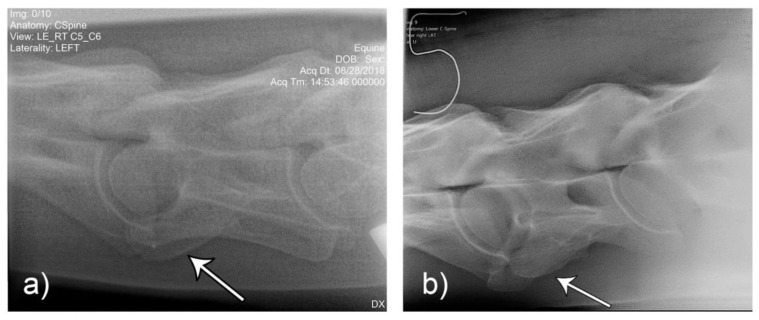
Laterolateral images F1 (**a**) and M2 (**b**) represent the malformation presentations of ECCMV: Unilateral (F1) and bilateral with (variable) bilateral transposition onto C7 (M2). Arrows indicate the abnormal line of the lamina in the absence of the caudal lamina of the transverse process.

Comparison of the unilateral and bilateral presentation variations of C6 was possible on the laterolateral views (Figure 10).

## 4. Discussion

Correct orientation and positioning are critical to reliably diagnose ECCMV radiographically. The equine cervical spine can be relatively easily palpated and methodically marked to aid in the accuracy of diagnosing lesions of ECCMV. On reviewing and taking the radiographs, the marker (positioned dorsal to the transverse process of C5) orientates the reviewer with respect to the C6 without obscuring the osseous architecture. Vertebrae C3 to C5 look similar on imaging with no distinguishing features so, the pre-placed marker is useful for correct identification of the preceding vertebrae. It also serves to orientate the operator when taking the radiographs.

Palpation of the cervical osseous landmarks can be improved with repetition but can be more challenging on heavily muscled or obese patients. The caudal aspect of the transverse process of C2 is smaller and slightly more medial to the transverse process of C3. However, the distance between these is closer than the following consecutive vertebrae. The cranial aspect of the transverse processes is much smaller and more medial and thus are more difficult to locate. C5 is the widest point on the equine cervical spine, and this is most easily located by palpating both transverse processes of C5 at the same time. Horses of heavier conformation and muscle mass may also prove more difficult to get exposure of the caudal laminae. In these cases, slight elevation of the head, placing the shoulder closet to the plate caudally, and turning the neck in slight lateral bend away from the generator may help present the lamina. Gentle cranial protraction of the neck can be achieved via a bilateral mandibular ascending ramus contact. Contacting the head or jaw cranial to the ascending rami leads to atlanto-occipital joint extension without lower cervical distraction.

Two common operator and reviewer errors are identification of a false bilateral morphologic variation of C6 when actually imaging the normal C5 vertebrae. Conversely, diagnosing the bilaterally absent ventral laminae on C6 as normal is another common mistake (Figure 10c). It should be noted that the caudal ventral tubercles of C6 have separate centers of ossification that may be misdiagnosed as fractures [7]. Asymmetry of the articular process joints in the cervical spine may make true laterolateral image capture difficult [7]. The laterolateral images of C6, along with the dorsal to ventral oblique images, are key to interpreting the symmetry, length, and shape of the lamina (Figure 4c). Field radiographs make the assessment of transposition on to C7 difficult in some cases. The transposition may be visible less commonly on the oblique views (Figure 9b) depending on the shape and position of the transposition [11]. Transposition on to C7 is more commonly seen on the laterolateral views and is usually seen below the transverse processes [11]. In some cases, the transposition may be seen on the oblique view (Figure 9b). Some three-dimensional alterations are so severe that misinterpretation of the morphology on C7 can occur (Figure 8c). This highlights the need for more detailed diagnostic assessment in these cases. Spinal cord compression by an asymmetrical canal, articular process periarticular bone formation and extent of intravertebral foramina compression cannot be assessed by radiography alone [10]. The study of the prepared vertebra in three dimensions is key to understanding the potential alterations in regional biomechanics.

Withers [11] has also described a lateral 45°–55° ventral-dorsal left and right views. This was not utilized in this protocol, as the ventral lamina and caudal ventral tubercle of C6 was not highlighted against the trachea, which aids in visualizing the whole shape and length of the lamina. This view may, however, be useful if the dorsal oblique view is, for some reason, not possible or difficult to obtain. The left and right 30° dorsal to ventral views, in conjunction with the laterolateral views of C6 and C7, are necessary for a diagnosis or exclusion of ECCMV (Figure 4). The potential issues arising from these osseous malformations and their subsequent altered muscular attachments can be appreciated on the vertebrae in cranial and ventral views (Figure 6a–c and Figure 7a–c).

Unpublished field research shows vertebral canal symmetry in ECCMV horses is often compromised in both C6 and C7 specimens [13]. The three-dimensional osseous changes associated with ECCMV [5] are likely to limit the usefulness of intravertebral sagittal ratios (IVRs), radiographs, and, indeed, myelography if the longitudinal symmetry of the vertebral canal in particular is affected. Further research and investigation may be warranted in cases that have been positively identified on radiography and have been refractory to other medical management and diagnosis. Subsequently, if this condition, with its altered muscle attachments, impacts lower cervical stability, then the risk factors for pain and dysfunction are likely to increase with age and trauma. To date, there are no longitudinal studies on clinically diagnosed patients assessing the progression of the dysfunction.

In the first author’s experience, scintigraphic examination has been of little value in identification of ECCMV. The condition is congenital, so it is unlikely to be recognized as a source of bone turnover. Computed Tomography (CT) is a modality that allows the three-dimensional assessment of these complex variations. However, due to its limited availability and expense to general client populations, the radiographic screening of cases prior to CT is of some clinical relevance. CT allows much more detailed assessment of transpositions of the ventral lamina, canal symmetry, altered arterial foramina, articular process joint integrity, and overall vertebral symmetry [10]. Skilled ultrasonography may also be of diagnostic and therapeutic use in these cases and may be important in assessing variations in osseous and soft tissue structures.

This study demonstrates that the positive identification of ECCMV via the addition of the 30° dorsal to ventral oblique views is possible. This allows identification of the condition in the live horse and provides a platform for further studies into its clinical relevance. Future studies with greater numbers of horses are needed.

## 5. Conclusions

The cervical region is sometimes overlooked as a source of pain and dysfunction in general equine veterinary practice. The advancement of radiographic technology and techniques mean that conditions such as ECCMV can now be reliably diagnosed using a standardized method. This allows further assessment and quantification of conditions that likely contribute to so called behavioral and training problems. Further research into the significance of ECCMV is needed to assess the potential impact on both horses, their welfare and their riders.
